# Bronchiolitis obliterans associated with toxic epidermal necrolysis induced by infection: A case report and literature review

**DOI:** 10.3389/fped.2023.1116166

**Published:** 2023-03-02

**Authors:** Jingwei Liu, Haibo Yan, Chunfeng Yang, Yumei Li

**Affiliations:** Department of Pediatric Intensive Care Unit, First Hospital of Jilin University, Changchun, China

**Keywords:** stevens-Johnson syndrome, dyspnea, toxic epidermal necrolysis, bronchiolitis obliterans, infection

## Abstract

**Background:**

Stevens-Johnson syndrome/toxic epidermal necrolysis has a severe impact on patients' eyes, genital mucosa, and many other organs. Bronchiolitis obliterans is a rare complication of Stevens-Johnson syndrome/toxic epidermal necrolysis.

**Data sources:**

We report a case of bronchiolitis obliterans associated with toxic epidermal necrolysis in our department. Furthermore, we examined the patients with bronchiolitis obliterans induced by Stevens-Johnson syndrome/toxic epidermal necrolysis and summarized the clinical characteristics, treatment, and prognosis. Databases available online in English including PubMed, Medline, and Web of Science were consulted.

**Results:**

We report one case and review 23 published case reports. Of the 24 patients, 13 were female, the oldest patient was 59 years old and the youngest was 5 years old. The time of bronchiolitis obliterans onset after Stevens-Johnson syndrome/toxic epidermal necrolysis varied from 5 days to 5 months. Bronchoscopy examination showed ulceration, exudative lesions, occlusion, and inflammation. The CT of lung manifestation included mosaic perfusion, bronchiectasis, consolidation, air trapping, pneumatocele, pleural thickening, lung collapse, larger central airway dilatation, lung overinflation, oligemia, and pneumomediastinum. Most cases indicated pulmonary function tests with obstructive ventilation dysfunction. The prognosis was poor; six of the patients died.

**Conclusions:**

Patients with Stevens-Johnson syndrome/toxic epidermal necrolysis may develop bronchitis obliterans at different stages, so all patients with Stevens-Johnson syndrome/toxic epidermal necrolysis should be followed up for possible respiratory complications.

## Introduction

Stevens-Johnson syndrome (SJS)/toxic epidermal necrolysis (TEN) are life-threatening dermatologic diseases characterized by the eruption of mucocutaneous blistering and epithelial sloughing (1). SJS and TEN are rare but are associated with many potential multisystem complications (2). Bronchitis obliterans(BO) causes obstruction and/or obliteration of the small airways, which is a chronic and irreversible obstructive lung disease (3). BO induced by severe lower respiratory tract infection is the most common form of BO in children (3). However, clinical reports about SJS/TEN complicated with BO are rare.

Herein, we report a case of BO associated with TEN in our department. Furthermore, we examined the patients with BO induced by SJS/TEN and summarized the clinical characteristics, treatment, and prognosis. This case report was approved by the Ethics Committee of First Hospital of Jilin University, China (2019-314). Informed consent was obtained from the parents of the patient. We reviewed relevant English literature from the online available databases, including PubMed, Medline, and Web of Science using the keywords “Stevens-Johnson syndrome”, “toxic epidermal necrolysis”, and “bronchiolitis obliterans”. The clinical characteristics, treatment, and prognosis of the participants in each study were summarized.

## Case presentation

**Figure 1:**
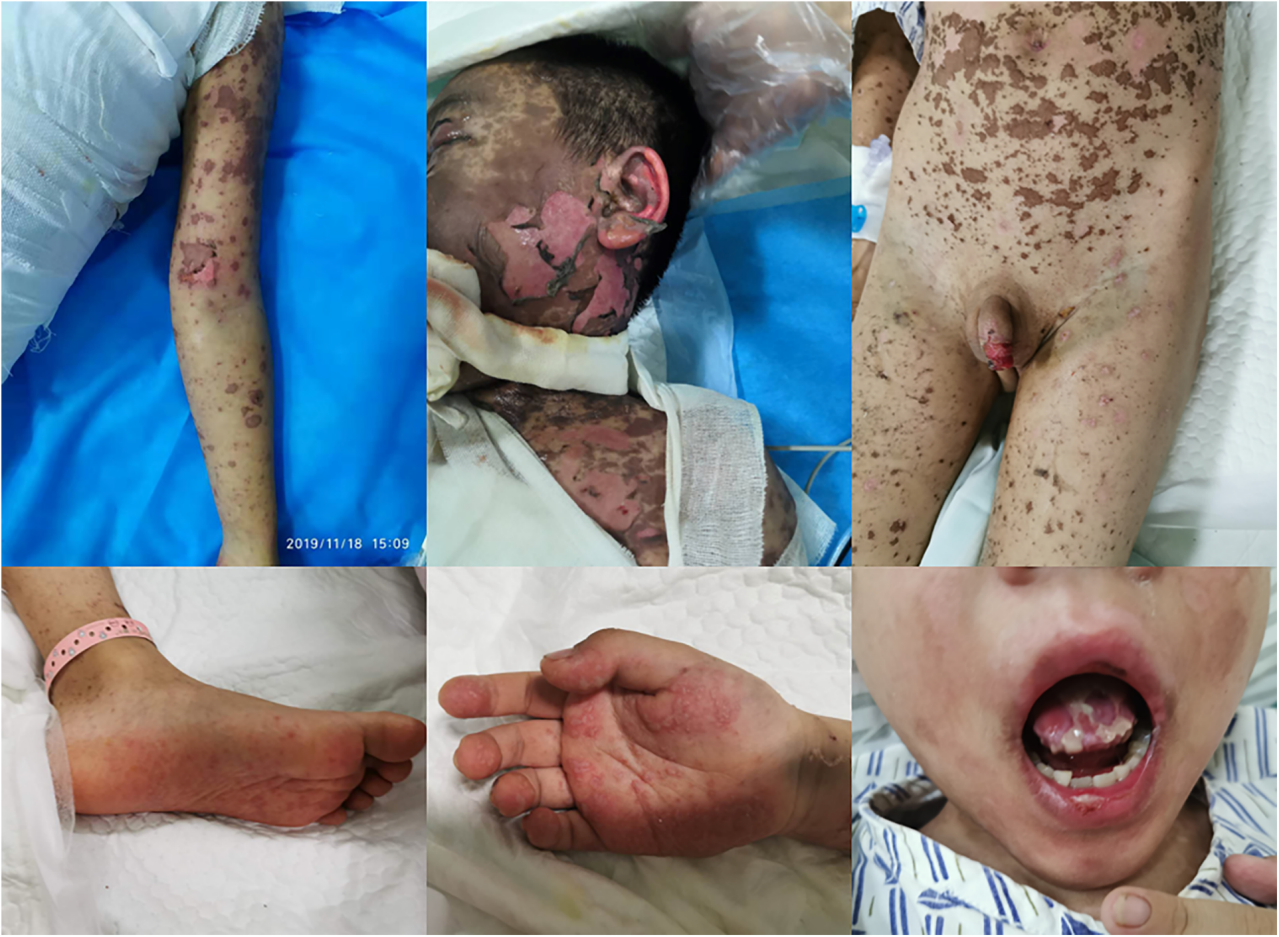
Rashes and blistering in different parts of the body.

**Figure 2:**
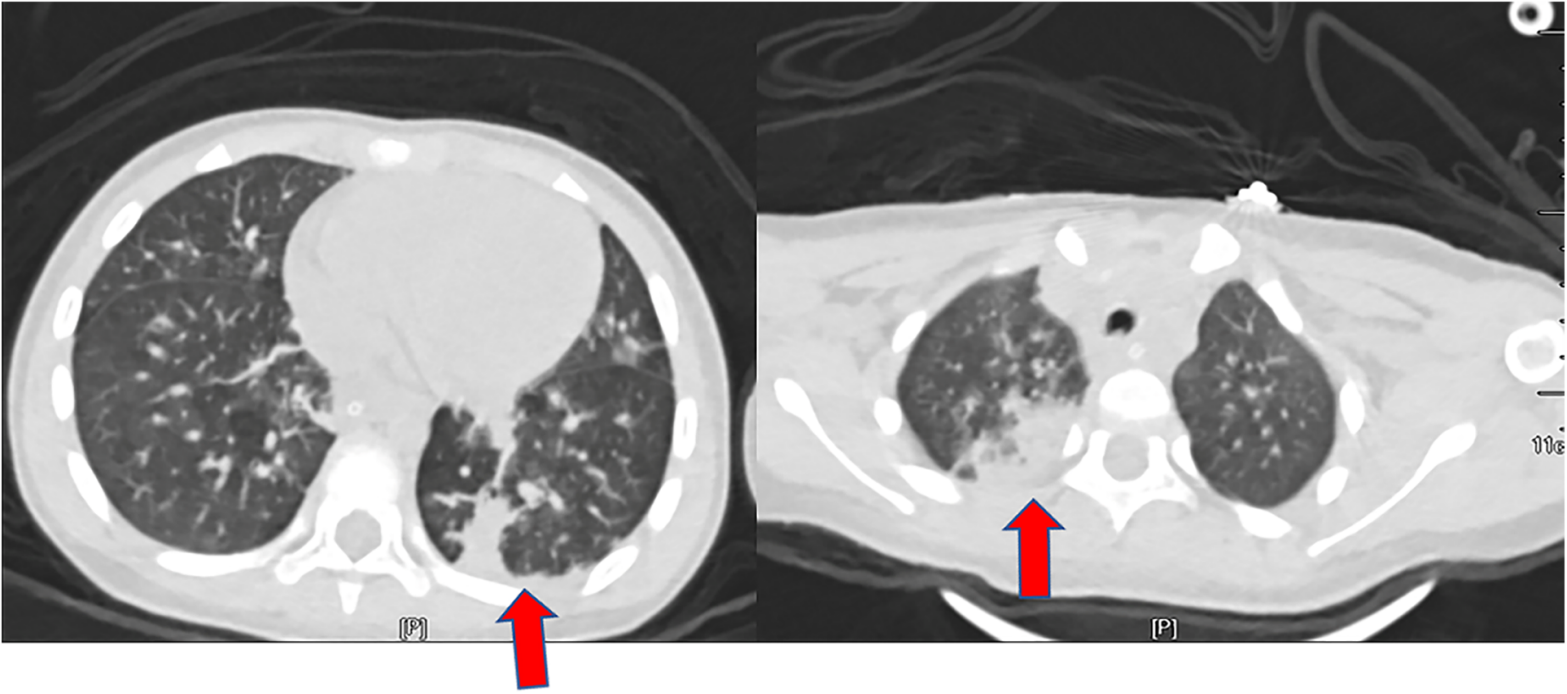
The CT scan of the chest performed 2 days after admission showed scattered consolidation.

**Figure 3:**
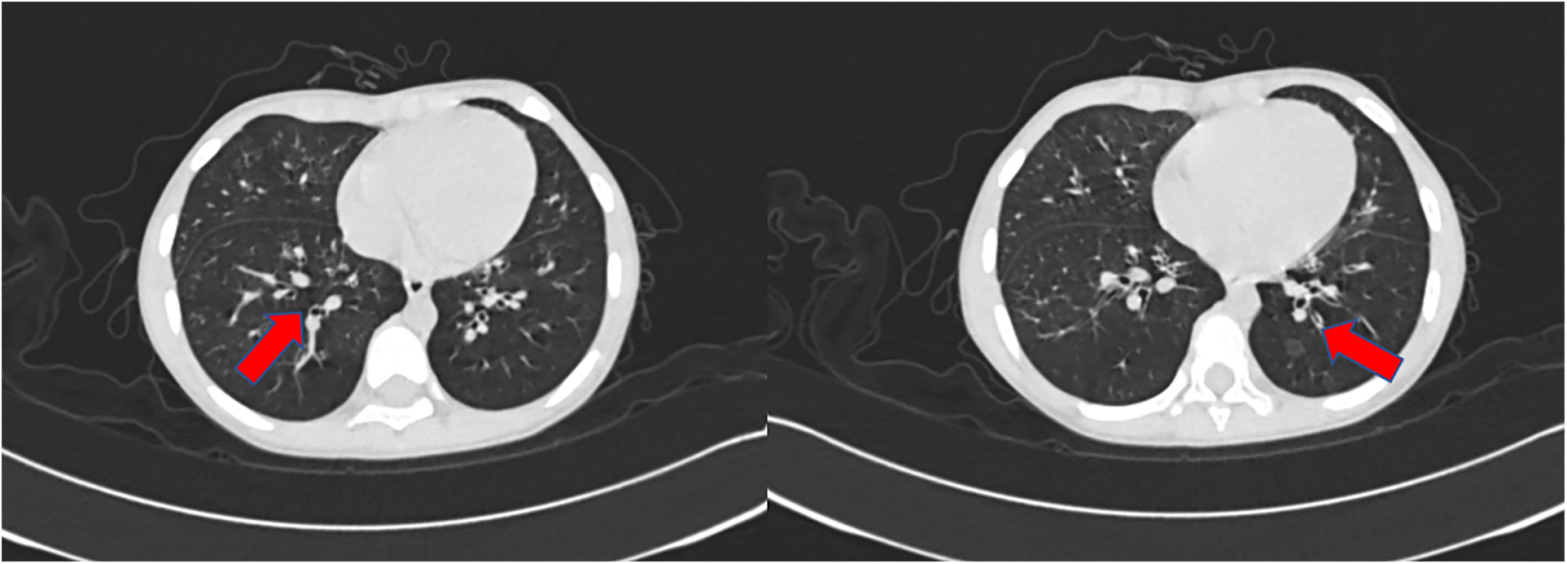
The CT scan of the chest performed 29 days after admission showed a thickening of airway walls.

**Figure 4:**
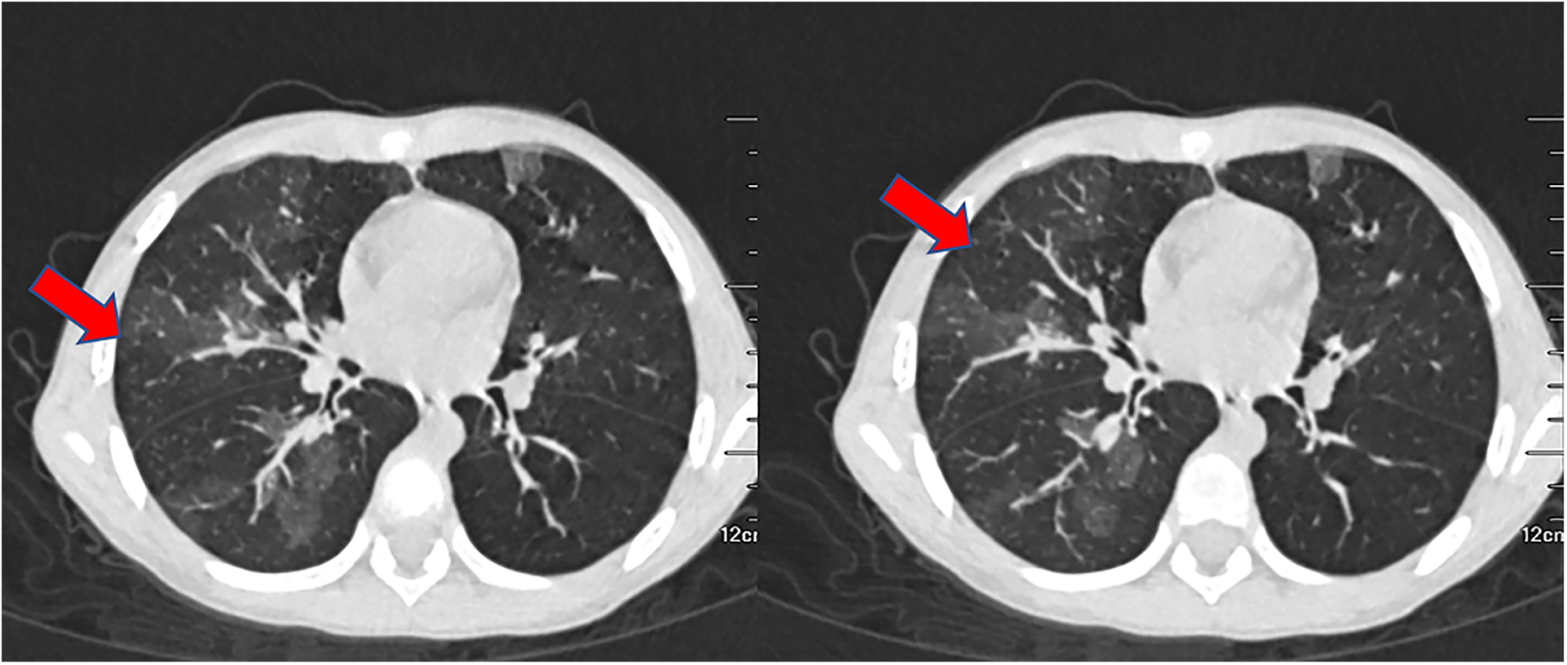
The CT scan of the chest performed 43 days after admission displayed widespread mosaic pattern.

A 6-year-old previously healthy boy presented to the emergency department of our hospital due to fever for three days, rash for two days, and lethargy for one day. The patient took oral antipathetic before admission and developed a rash before taking antipathetic. He was transferred to the pediatric intensive care unit for further treatment. Upon admission, vital signs revealed temperature at 39°C, pulse rate at 182 beats/min, respiratory rate at 40 breaths/min, and blood pressure at 93/53 mm Hg. He also had a diffuse dark red rash and vesiculobullous lesions involving his face, ear, trunk, and extremities (>30% of the body surface area) (Figure 1). Parts of the rash and blister were broken with serous exudates. The boy could not open his eyes, which were covered with many yellow secretions. The conjunctiva of both lower eyelid, lip, tongue, penile mucosa, and oral mucosa were broken. Auscultation of the two lungs showed some rales. Laboratory tests reflected normal leukocyte count, elevated C reactive protein (112.7 mg/l), and procalcitonin (89.97 ng/ml). Serum cytokine concentration showed that the serum IL-6 was 1,552.24 pg/ml and the serum IL-10 was 135.69 pg/ml. Other laboratory findings were as follows: Mycoplasma pneumoniae (MP) IgM 1.55 COI, MP IgG 237.00 AU/ml, creatine kinase 1,470 U/l, creatine kinase isoenzyme 161.1 U/l, lactate dehydrogenase 778 U/l, aspartate aminotransferase 108 U/l, alanine aminotransferase 35.9 U/l, urinary protein 2+, urinary RBC count 32.0/µl, IgE 764.00 IU/ml, serum ferritin 474.6 ug/l, D-dimer 2.17 ug/ml. The lung CT showed scattered consolidation two days after admission (Figure 2). Upon admission, treatment with invasive mechanical ventilation, systemic steroid therapy, IVIG, azithromycin, vasopressor, and topical medications of eye, and skin dressing were initiated immediately to rescue the patient. Three days after admission, laboratory investigations worsened progressively as follows: creatine kinase 21,098 U/l, creatine kinase isoenzyme 434 U/l, lactate dehydrogenase 1,622 U/l, aspartate aminotransferase 823.5 U/l, alanine aminotransferase 196.2 U/l, amylase 2,069 U/l, C reactive protein (225 mg/l). Due to severe inflammatory reactions and multisystem complications, continuous blood purification was started. Seven days after admission, vital signs and laboratory investigations improved progressively, so continuous blood purification and invasive mechanical ventilation were all removed. The respiratory status was normal without cough, dyspnea, and wheezing, and auscultation of the two lungs showed no rales. The patient's skin and mucosa lesions also improved gradually. Methylprednisolone was gradually decreased from 2 mg/kg/day to 0.5 mg/kg/day and finally stopped. Methylprednisolone was used for 10 days in all. Methylprednisolone was used for 10 days in all. In spite of the improvement of the skin and mucosa lesions, the patient began to suffer from cough and slight tachypnea 26 days after admission. The lung CT showed thickening of the airway wall of two lungs without atelectasis or pneumonic consolidation 29 days after admission (Figure 3). The patient was treated with budesonide, bronchodilators atomization inhalation, and antibiotics. Pulmonary function tests on day 33 revealed extremely severe obstructive dysfunction with forced vital capacity (FVC) of 0.48 L (28.2% predicted), forced expiratory volume in 1 s (FEV1) of 0.28 L (19.4% predicted), FEV1/FVC ratio of 67.8% predicted. Bronchodilation test was negative. The thorax CT showed a widespread mosaic pattern 43 days after admission (Figure 4). The patient developed obvious wheezing and progressive dyspnea with poor response to bronchodilators, so nasal oxygen inhalation and oral prednisone were started. He was discharged home without tachypnea at rest 56 days after admission. After discharge, the patient took prednisone for 2 months and inhaled budesonide for more than 2 years. Four months after discharge, pulmonary function tests revealed severe obstructive dysfunction with FVC of 0.59 L (36.2% predicted), FEV1 of 0.35 L (25.8% predicted), FEV1/FVC ratio of 70.2% predicted. The patient had no wheezing when in a quiet state or undertaking slight activity but could not tolerate intense activities.

## Discussion

SJS, SJS/TEN overlap and TEN are different due to the degree of skin detachment: the skin detachment area of SIS < 10%, the skin detachment area of TEN > 30%, the skin detachment area of SJS/TEN overlapping is 10%–30% (4). The causes of SJS/TEN include infection, drugs, and immunity (4). MP infection is associated with SJS/TEN (5). An investigation into outbreaks of MP-associated SJS revealed 3-X-6-2 MP strain is more common in SJS patients than patients with pneumonia only (6). Clinical manifestations of MP-associated SJS include increased erythrocyte sedimentation rate, respiratory infection, and less extensive skin lesions (7, 8). Our patient had a rash before taking medicine, the laboratory data related to infection was significantly increased above normal and the IgM of MP was positive. In this case, we reported that the infection might be the offending agent of the patient, according to medical history and auxiliary examination.

The interventions in the British Association of dermatologists' guidelines for the management of SJS/TEN include corticosteroids, IVIG, ciclosporin, low molecular weight heparin, biological therapy, granulocyte-colony stimulating factor, calcineurin inhibitors, and antibiotics (9). The symptoms of mucocutaneous blistering and epithelial sloughing gradually improved after the patient was treated with methylprednisolone and IVIG.

SJS/TEN has a severe impact on the eyes, kidney, genital mucosa, and other organs (4). Our patient developed renal injury, myocardium injury, liver injury, pancreatic injury, and ophthalmic complications. In the acute phase, nearly 40% of patients with SJS/TEN developed respiratory complications (1, 10). The respiratory involvements include the exfoliation of bronchial epithelium, pulmonary edema, atelectasis, and infectious pneumonia. The late sequelae of SJS/TEN survivors revealed interstitial lung disease, airway obstruction, bronchiectasis, bronchitis, and BO (1). BO is an uncommon complication of SJS/TEN (11). On reviewing the literature, we discovered 23 cases regarding BO associated with SJS/TEN in children and adults (Table 1). In the 23 published cases, 13 patients were female, the oldest aged 59 years and the youngest aged 5 years. Physical examination of BO shows tachypnoea, crackles, and persisting hypoxemia (3).

**Table 1:** Summary of clinical manifestation in BO^1^ associated with SJS^2^/TEN^3^ from the published cases.

No.	Age	Sex	Cause of SJS/TEN	Pathogen	BO onset time^4^	PFT^5^	Chest CT	Bronchoscopy	Reference
1	8	Female	MP^6^	MP	1 month	Unknown	Unknown	Main and segmental airways were normal.	(12)
2	41	Female	Ampicillin, cephamandole, anti-inflammatory agents	Unknown	24 days	Unknown	Unknown	Unknown	(13)
3	25	Male	Phenytoin	Unknown	Unknown	Obstructive dysfunction	Unknown	Occlusion of the of right B9 bronchus	(14)
4	10	Male	Rifampin, pyrazinamide, isoniazid	Unknown	2 months	Unknown	Mosaic perfusion, bronchiectasis, consolidation	Unknown	(15)
5	6	Female	Ampicillin, amoxicillin, acetaminophen	Unknown	5 days	Unknown	Air trapping, mosaic perfusion.	Unknown	(15)
6	13	Male	Cefcapene pivoxil hydrochloride, amantadine hydrochloride	Unknown	7 days	Unknown	Parenchymal lung disease,pneumatocele.	Ulcerative and exudative lesions with sloughing of mucosa throughout the respiratory tree.	(16)
7	8	Male	Benzathine penicillin	Unknown	14 days	Oobstructive dysfunction	Bilateral mosaic pattern	Unknown	(17)
8	13	Male	Cefazolin sodium	Unknown	5 months	Obstructive dysfunction	Mosaic pattern	Unknown	(17)
9	6	Female	Unknown	Unknown	Unknown	Unknown	Unknown	Unknown	(18)
10	6	Male	Unknown	Unknown	Unknown	Unknown	Unknown	Unknown	(18)
11	6	Female	Medication	Unknown	14 days	Unknown	Unknown	Unknown	(19)
12	5	Male	Nimesulide	Unknown	14 days	Unknown	Mosaic pattern, collapse,consolidation	Unknown	(20)
13	25	Female	Amoxicillin	Unknown	2 months	Mixed ventilatory and small airways impairment	Mosaic pattern, air trapping, pleural thickening,	Unknown	(21)
14	9	Female	Lamotrigine	Unknown	7 days	Obstructive dysfunction	Right lung collapse,larger central airway dilatation, left lung over inflation.	Occlusion of the right B4b bronchus	(22)
15	59	Male	Unknown	Unknown	2 months	Obstructive dysfunction	Bronchial dilatation, oligemia, air trapping	No endobronchial lesions	(23)
16	8	Female	Paracetamol and nimesulide	Unknown	1 month	Obstructive dysfunction	Pneumomediastinum	Normal	(24)
17	11	Female	Clarithromycin	Unknown	Not described	Unknown	Not described	Unknown	(2)
18	41	Female	Cefuroxime	Unknown	3 months	Obstructive dysfunction	Bronchiectasis,mosaic attenuation.	Unknown	(25)
19	5	Male	Infection	Pseudomonas aeruginosa adenovirus rhinovirus enterovirus	5 months	Obstructive dysfunction	Mosaic perfusion, air trapping	Ulceration at the base of insertion of the endotracheal	(11)
20	5	Male	Adenovirus	Adenovirus.	1 month	Obstructive dysfunction	Air trapping, vascular paucity.	Unknown	(11)
21	14	Female	MP	MP	1 month	Obstructive dysfunction	Confirmed BO	Unknown	(11)
22	7	Female	Not described	Not described	1 month	Obstructive dysfunction	Mosaic pattern,air trapping, bronchiectasis	Intense mucosal inflammation of airways	(26)
23	10	Female	Paracetamol ibuprofen	Unknown	1 month	Unknown	Air trapping with perfusion defects, bronchial wall thickening and bronchiectasis	Unknown	(27)

^1^ BO, Bronchiolitis obliterans.

^2^ SJS, Stevens-Johnson Syndrome.

^3^ TEN, toxic epidermal necrolysis.

^4^ BO onset time, Time interval between SJS/TEN and BO.

^5^ PFT, Pulmonary function test.

^6^ MP, Mycoplasma pneumoniae.

The mechanism of BO secondary to SJS/TEN remains unclear; it may be that the immune complex deposition results in the damage of bronchial epithelial cells and mucosa (21). The combination of abnormal immune response and respiratory infection may play an important role in the occurrence of BO in SJS/TEN patients (21). Table 1 shows four patients infected. Our patient's MP IgM was positive. MP infection has a higher risk of development for post-infectious BO (28). BO may occur following acute MP bronchiolitis due to airway epithelial injury and sloughing (29). Hypoxemia and high level of lactate dehydrogenase are the risk factors for BO in children with MP Bronchiolitis (30, 31). Our patient had hypoxemia and a high level of lactate dehydrogenase. MP infection may be one of the reasons for the development of BO in our patient. Evidence of infection was also identified in patients with BO associated with SJS/TEN (11, 12). It is unclear whether MP infection is a cofactor for BO secondary to SJS/TEN or just an etiological factor in patients with SJS/TEN (1).

Autopsy of BO associated with SJS/TEN showed diffuse epithelial shedding and partial regeneration of the tongue, pharynx, and trachea (13). Eight of the published cases of BO-associated SJS/TEN provided bronchoscopy results. At the early stage of SJS/TEN, bronchoscopy examination showed ulceration and exudative lesions with mucosal detachment in the whole respiratory tract (16). Bronchoscopy of other published cases showed occlusion of the bronchus, no endobronchial lesions, normal main and segmental airways, ulceration at the base of insertion of the endotracheal, and intense mucosal inflammation of airways (11, 12, 14, 22–24, 26). Our patient did not complete a bronchoscopy examination, this is a limitation of this case.

Lung biopsy is regarded as the gold standard for the diagnosis of BO. Due to the patchy distribution of BO, it is difficult to obtain tissue with characteristic pathological changes (3). The clinical diagnosis of BO was made on the basis of clinical characteristics, pulmonary function examination results, and the typical HRCT manifestations (29). According to the persistent respiratory manifestation, pulmonary CT scan, and pulmonary function test of our patient, BO was diagnosed. The CT of lung manifestation included mosaic perfusion, bronchiectasis, consolidation, air trapping, pneumatocele, pleural thickening, lung collapse, larger central airway dilatation, and lung overinflation. oligemia, pneumomediastinum as shown in Table 1. Most of the published cases indicated pulmonary function tests with obstructive ventilation dysfunction. We also summarized the time of BO onset after SJS/TEN in Table 1. Some patients developed a productive cough and dyspnea 5 days after the appearance of SJS (15). So far the longest time between the onset of respiratory symptoms and initial presentation with SJS is 5 months (17). Even if there are no respiratory symptoms in the early stage, we should closely monitor the development of BO for a long time.

The modalities that have been used in the treatment of BO include azithromycin (32), steroids (33), extracorporeal photopheresis (34), rituximab (35), lung transplantation (36), and so on. Our patient showed improvement in respiratory symptoms and daily activities, after using systemic steroids and azithromycin. We summarized the treatment and outcome of the previous cases as shown in Table 2. All patients received steroid therapy, four patients underwent lung transplants, some patients received bronchodilators, some used macrolides, and some patients received immunosuppressive agents. Owing to post-SJS/TEN, BO is progressive and irreversible; azathioprine can be used in refractory cases (37). There is no clear treatment strategy for airway mucosal diseases and long-term steroid therapy may cause secondary pulmonary infection; therefore, further research is needed (38). A case report showed that the SJS patient who developed severe symptoms of BO did not receive immunomodulatory or systemic immunosuppressive therapy in the acute phase (11). Continuous blood purification could ameliorate the inflammatory response (39). A retrospective cohort study revealed continuous venovenous hemofiltration combined with hemoperfusion might be an effective and safe adjuvant therapy for TEN (40). A drastic decrease was observed in the level of IL-6 and IL-10 after continuous blood purification therapy in our patient. Although we started systemic corticosteroids, continuous blood purification, and IVIG in the acute stage, we still can not protect our patient from developing BO. Whether early intervention would have an impact on the development of BO in SJS/TEN is unclear. More research is needed to reduce the risk of SJS/TEN leading to BO.

**Table 2:** Summary of treatment and prognosis in BO^1^ associated with SJS^2^/TEN^3^ from the published cases.

No.	Age	Sex	Treatment for SJS/TEN	Treatment for BO	Prognosis	References
1	8	Female	Erythromycin	Steroid,bronchodilators	Die after 10 months	(12)
2	41	Female	Steroid	Steroid, bronchodilaters	Die after 2 months	(13)
3	25	Male	steroid	Steroid, bronchodilaters, erythromycin	Alive after 1 years	(14)
4	10	Male	Unknown	Steroid, bronchodilators, antibiotics. oxygen inhalation, physiotherapy	Alive after 21 months	(15)
5	6	Female	Unknown	Steroid, bronchodilators, antibiotics, physiotherapy	Alive after 27 months	(15)
6	13	Male	Steroid	Lung transplantation	Alive after 11 months	(16)
7	8	Male	Unknown	Steroid, bronchodilators, oxygen inhalation, physiotherapy, antibiotic	Alive after 15 months	(17)
8	13	Male	Unknown	Steroid, bronchodilators, oxygen inhalation, physiotherapy	Alive after 8 months	(17)
9	6	Female	Unknown	Steroid	Alive	(18)
10	6	Male	Unknown	Steroid, lung transplantation	Die	(18)
11	6	Female	Unknown	Lung transplantation	Alive after 9 months	(19)
12	5	Male	Unknown	Steroid, bronchodilators, azathioprine	Died after 1 year	(20)
13	25	Female	Unknown	Steroid, azithromycin	Die after 17 years	(21)
14	9	Female	Steroid	Steroid, bronchodilators, bronchoalveolar lavage, physiotherapy, antibiotics	Alive after 10 months	(22)
15	59	Male	Steroid	Steroid, bronchodilators, roxithromycin, tracheostomy, ventilation	Died after 6 months	(23)
16	8	Female	IVIG	Steroid	Alive after 9 months	(24)
17	11	Female	Unknown	Unknown	Unknown	(2)
18	41	Female	steroid	Steroid, bronchodilators, azithromycin	Alive after 10 years	(25)
19	5	Male	IVIG, steroid, mycophenolate mofetil, ciclosporin.	Steroid, azithromycin, physiotherapy	Alive after 1 years	(11)
20	5	Male	IVIG^4^	Steroid, azithromycin, physiotherapy	Alive	(11)
21	14	Female	Azithromycin, steroid	Steroid, azithromycin, ciclesonide, physiotherapy	Alive after 6 months	(11)
22	7	Female	IVIG	Steroid, azithromycin, lung transplantation	Alive after 3 years	(26)
23	10	Female	Corticosteroids and cyclosporine	Lung transplantation	Alive after 3 years	(27)

^1^ BO, Bronchiolitis obliterans.

^2^ SJS, Stevens-Johnson Syndrome.

^3^ TEN, toxic epidermal necrolysis.

^4^ IVIG, Intravenous immunoglobulin.

Although the understanding of the pathogenesis, diagnosis, and treatment of BO have made some progress in the past years, the overall mortality is still very high (41). The prognosis of BO is variable and depends on the initial cause.

BO associated with SJS/TEN is progressive and has a poor prognosis (27). Six of the case reports ended in death, as shown in Table 2. A case report described BO complicating a pneumothorax after SJS (22). Some reported cases showed that death occurs due to respiratory failure, and the longest recorded survival time was 17 years after SJS (21). The shortest recorded survival time was 2 months after SJS (13).

## Conclusion

BO secondary to SJS /TEN is a rare but devastating disorder. Continuous monitoring and timely treatment of SJS/TEN contribute to preventing the progression of the disease. Patients with SJS/TEN may develop BO at different stages, so we should follow all patients with SJS/TEN for possible persistent respiratory complications for as long as possible even after they recover from SJS/TEN. In this area, further research is required to explore potential mechanisms and develop better monitoring pathways and treatment options.

## Data Availability

The original contributions presented in the study are included in the article/Supplementary Material, further inquiries can be directed to the corresponding author/s.
